# Annotations

**Published:** 1896-05-02

**Authors:** 


### Annotations.

### The Last of Quarantine in England.

A Bill is now before Parliament to abolish the last trace of quarantine in England. Long before the days of sanitary legislation, in the modern acceptation of the term, laws were passed in many countries with the object of preventing the importation of diseases from abroad. In early times these applied principally to plague against which quarantine regulations have been common in the South of Europe ever since the middle of the fourteenth century. It is a curious thing that plague had disappeared from England for more than thirty years before the practice of quarantine against it was definitely established by Act of Parliament in 1710, and that, with the exception of yellow fever, plague still remains, the one disease against which the provisions of the Quarantine Act, now to be repealed, remains in operative activity. Yellow fever, however, stands in a somewhat different category. Plague had practically been forgotten when yellow fever became a danger to Europe. During the eighteenth century several outbreaks occurred in Spain, the disease being mostly limited to Cadiz. But in the early years of this century it spread largely over the Peninsula, causing a very considerable mortality, and much anxiety was felt lest it should gain a footing in England. In 1825, then, was passed the Quarantine Act, according to which yellow fever or other highly infectious disorders were recognised as calling for quarantine along with plague. Probably it was this fact that yellow fever and plague were looked upon as already provided for, which led to their special exclusion from the list of those diseases which were required to be reported by the Customs officers to the local sanitary authorities when these came into existence; and so it happened that, while the sanitary administration of the country was dealing with every other kind of malady, the responsibility of taking precautions against these two diseases continued, and still continues, to devolve upon the Imperial Government, which has neither quarantine establishments nor isolation hospitals for the purpose. So far as plague is concerned this does not matter, but yellow fever is endemic in ports with which we have considerable trade, and it was imported into Swansea ln 1865, when it caused a considerable mortality. It is high time, therefore, that its control should be handed over to the sanitary authorities.

### Is Typhoid a Preventable Disease?

Any man or woman who has ever struggled through the ten, twelve, or fifteen weeks' experience of a determined attack of typhoid fever will think we have placed an exceedingly important question at the head of this annotation. Can typhoid be prevented? Well, either it can or it cannot; either it is a disease whose cause we know and can deal practically with, or it remains still one of those mysteries which baffle science and practice alike. But typhoid is not now a "mystery" to the man of science; of that we are sure. It is, on the contrary, a well understood, and entirely manageable and preventable malady. Dr. T. E. Hayward, medical officer of health for Haydock, Lancashire, tells his parish in his annual report not only that it is preventable, but also by what precise and simple means it may be prevented. We have had occasion before to express approval of Dr. Hayward's intelligence and thoroughness as a medical officer of health, and we commend with confidence his tersely expressed "Principles of Typhoid Prevention," as made public in his report for 1895. "Typhoid is a preventable disease," says this authority. "It is a disease of filth, and especially of the filth of human excrement." That is to be taken as the beginning of knowledge, the A B C of typhoid prevention, by the unscientific. The next step is equally easy. Typhoid is a disease of bacilli, typhoid bacilli; and the bacilli we find in human excrement, and in drinking water fouled by such excrement. It is probable that no man or woman ever takes typhoid except by swallowing some typhoid bacilli. They may be swallowed, as we have said, in drinking water, or they may be flying about in the air in the neighbourhood of typhoid excrement, and may be swallowed with mouthfuls of air. What then is the first and last commandment of typhoid prevention? "Cleanliness; personal and public cleanliness." It is all there. If we keep ourselves clean; if we keep our drinking water clean; if we keep our closets and our drains, our kitchens, sculleries, gardens, streets, and towns entirely clean, typhoid will be practically as great a stranger to most of us as is the ghost of King Solomon or the shade of the extinct Deinotherium.

### The Microbe in Daily Life.

At a meeting held at Glasgow the other day, under the auspices of the sanitary authorities, to hear a lecture on the subject of microbes, by Professor Glaister, M.D., such was the conviction which the lecturer's reasoning carried to the minds of his audience that they resolved forthwith, on the motion of Bailie Crawford, to institute a "Department ot Bacteriology" in connection with the sanitary work of the city. The great enemy to the public health in large cities, say the Glasgow sanitarians, is the "microbe," and if a successful war is to be waged against him the first condition of such warfare is to obtain a full knowledge of him, his methods of attack, and the kinds of defence and counter-attack which are likely to be most successful in meeting and annihilating him. The busiest city in Scotland is well aware of the proud pre-eminence in science and sanitation which she is thus securing for herself. As Bailie Crawford enthusiastically claimed, she is going "to take a step in advance of all the municipalities of the kingdom" in thus bringing the microbe within the activities of everyday life. We have only one word of criticism to utter. If Glasgow does not become healthier and show a lowered death-rate in consequence of this opening out of the microbial region to common life, her sanitarians and plumbers must face the ridicule they will not fail to encounter. If they enter upon a campaign against the microbe they must do it thoroughly. Money, spent freely, will secure the best services which science and practice have at disposal. Let the Glasgow authorities spend money enough, and we venture to promise them a success which will amply justify their excellently-intended new sanitary departure.

